# Alcohol Screening and Brief Intervention in Workplace Settings and Social Services: A Comparison of Literature

**DOI:** 10.3389/fpsyt.2014.00131

**Published:** 2014-10-06

**Authors:** Bernd Schulte, Amy Jane O’Donnell, Sinja Kastner, Christiane Sybille Schmidt, Ingo Schäfer, Jens Reimer

**Affiliations:** ^1^Centre for Interdisciplinary Addiction Research, University Medical Centre Hamburg-Eppendorf, Hamburg University, Hamburg, Germany; ^2^Institute of Health and Society, Newcastle University, Newcastle, UK

**Keywords:** brief alcohol intervention, workplace health, social services, criminal justice setting

## Abstract

**Background:** The robust evidence base for the effectiveness of alcohol screening and brief interventions (ASBIs) in primary health care (PHC) suggests that a widespread expansion of ASBI in non-medical settings could be beneficial. Social service and criminal justice settings work frequently with persons with alcohol use disorders, and workplace settings can be an appropriate setting for the implementation of alcohol prevention programs, as a considerable part of their social interactions takes place in this context.

**Methods:** Update of two systematic reviews on ASBI effectiveness in workplaces, social service, and criminal justice settings. Review to identify implementation barriers and facilitators and future research needs of ASBI in non-medical settings.

**Results:** We found a limited number of randomized controlled trials in non-medical settings with an equivocal evidence of effectiveness of ASBI. In terms of barriers and facilitators to implementation, the heterogeneity of non-medical settings makes it challenging to draw overarching conclusions. In the workplace, employee concerns with regard to the consequences of self-disclosure appear to be key. For social services, the complexity of certain client needs suggest that a stepped and carefully tailored approach is likely to be required.

**Discussion:** Compared to PHC, the reviewed settings are far more heterogeneous in terms of client groups, external conditions, and the focus on substance use disorders. Thus, future research should try to systematize these differences, and consider their implications for the deliverability, acceptance, and potential effectiveness of ASBI for different target groups, organizational frameworks, and professionals.

## Background

Alcohol is a significant risk to public health (1) and globally, heavy drinking represents the fifth leading cause of morbidity and premature death after high-blood pressure, tobacco smoking, household air pollution from solid fuels, and a diet low in fruits (2). A variety of interventions exist for the prevention and treatment of alcohol-related risk and harm, ranging from health promoting interventions aimed at tackling hazardous and harmful drinking, to more intensive and specialist treatment for severely dependent drinking (3). Alcohol screening and brief intervention (ASBI) has emerged as an effective, and cost-effective, preventative approach to reduce hazardous, and harmful drinking in non-treatment seeking individuals, and has been shown consistently to reduce the quantity, frequency, and intensity of drinking when delivered in primary health care (PHC) settings (4).

The robust evidence for ASBI effectiveness in PHC suggests that an extension of ASBI implementation into further settings with groups that may be at an increased risk of alcohol-related harm may be beneficial (5). For example, while the evidence remains equivocal, individual studies have demonstrated positive effects of ASBI in emergency departments and general hospital wards (6, 7). Also, non-medical settings may also provide a valuable point of contact to risky drinkers (5), and to target groups who are not routinely accessed via PHC settings. Not least, as in addition to the well documented health harms (2), alcohol also impacts significantly upon individuals, families, and communities, with heavy drinkers potentially experiencing social harms such as family disruption, interpersonal violence (8–10), involvement in crime, problems within the workplace, and financial difficulties (11).

First, social work has a long history of working with persons with alcohol or substance use disorders (12, 13), and therefore, social services in their various forms potentially represent an important field for brief intervention delivery. In an US survey on a large and representative sample of social workers, 71% of respondents reported having taken some action related to substance abuse diagnosis and treatment in the preceding 12 months with clients, whereas only 2% stated substance use disorders being their primary practice area (14). Indeed, further studies confirm the substantial contribution of substance misuse to a social worker’s caseload in children’s services (8), mental health services (15), adult’s services (16), as well as those employed within specialist drug and alcohol teams (17). Importantly, delivering ASBI within a social service setting may take advantage of the teachable moment wherein individuals can consider their alcohol use behavior within the context of the contact with a social worker: an approach, which has been shown to be beneficial within PHC (18). Thus, social service and criminal justice system settings may be another valuable point to contact further populations of risky and hazardous drinkers who are not necessarily reached within healthcare settings.

Second, given that alcohol is the most widely used substance among working adults, and the fact that almost 80% of risky drinkers are employed, workplace health services may also present a valuable opportunity for the delivery of preventative alcohol work. Alcohol abuse is associated with multiple negative workplace outcomes, including absenteeism, accidents, turnover, and other sources of productivity losses (19–23). Specific job-related influences associated with problem drinking, including job stressors and participation in work-based drinking networks, may pose a particular problem for young adults as they try to fit in their workplace (24). Using the workplace for the provision of alcohol prevention is important because the workplace is an identifiable setting where a prevention program can be disseminated (25). Further, the workplace is a traditional setting for providing prevention messages to individuals with drinking problems (26), and therefore, a useful existing network in which health psychologists, behavioral medics, public health professionals, and employers can deliver health-related messages and interventions regarding alcohol consumption that reach the majority of employees (27). Workplaces also appear to be appropriate sites for conducting early interventions as most people spend substantial periods of time at work (26, 28). For example, 28% of the 18 million salaried French people who are looked after by their occupational health doctor see no other doctor during the year (29).

Against this background, this paper examines the existing evidence for the delivery of ASBI in social service and workplace settings, and considers the challenges that providers and recipients alike might experience in achieving their routine implementation. In doing so, we report on the findings of two recent setting-specific (social services and workplace) systematic literature reviews focused around three key questions:

1. First, what evidence is there for the effectiveness of ASBI in social service and workplace settings?
2. Second, what barriers and facilitators exist to ASBI implementation in social service and workplace settings?
3. Third, and finally, what are the key evidence gaps and future research needs in this area of ASBI research?

The present study aimed to update the results obtained in a previous search^1^ conducted as part of the European Union financed BISTAIRS research project. Additionally, we expanded the original research question by adding the analysis of barriers/facilitators to ASBI implementation and by reviewing the need for future research in these settings.

## Methods

The following electronic databases were searched: Medline (OVID); EMBASE (OVID); PsycInfo (OVID); The Cochrane Library (Wiley); CINAHL (EBSCO); and Web of Science (Databases: SCI-EXPANDED, SSCI, A&HCI) using appropriate MeSH terms. The search was divided into three core concepts:

- Setting: workplace, worksite, occupational, employee, or labor; social service, social work, services for homeless people, employment agencies, non-scholar youth work, criminal justice, and probation/rehabilitation services (including interventions for traffic offenders under the influence of alcohol), and community-based institutions, e.g., (drug) counseling centers;
- Intervention: alcohol, brief intervention, alcohol therapy, counseling, and early intervention; and
- Study design: primarily randomized controlled trials (RCTs).

Additional information and further sources obtained from experts in the field and websites of relevant organizations/networks and reference lists of included articles were considered. The selection of studies comprised, in a first step, screening of title and abstract, which was also achieved by identifying keywords for exclusion. Second, for potentially relevant articles, the full text was retrieved and examined in-depth against a detailed set of inclusion criteria.

Studies on the effectiveness of brief alcohol intervention in comparison to control conditions, which were delivered in either workplace or social service settings, and published between January 2002 and June 2013 in English, were eligible for inclusion. Primarily, we aimed to include RCTs and also searched for prospective observational studies to consider them subordinately, as an initial scoping search suggested that only a small number of RCTs in social service and workplace settings would be identified. ASBI was defined as a single session or up to a maximum of four sessions of engagement with a client or employee and the provision of information and advice that is designed to achieve a reduction in risky alcohol consumption or alcohol-related problems. Studies with single sessions longer than 40 min were excluded. Brief interventions were typically compared to control conditions of assessment only or treatment as usual.

Primary outcomes of interest included changes in self- or other-reports of drinking quantity and/or frequency, drinking intensity (e.g., number of drinks per drinking day), and drinking within recommended limits. Risky drinking was defined as drinking in excess of 60 g of alcohol per day for men and 40 g for women (30). Hazardous drinking is consumption at a level, or in such a pattern, that increases an individual’s risk of physical or psychological consequences (31), while harmful drinking is defined by the presence of these consequences (32). While the concept of workplace setting is relatively well defined, the definition of the setting “social services” is more ambiguous. We included studies based in the following settings or populations: homeless people, offenders under the influence of alcohol, youth work/youth welfare services, employment agencies, and (drug) counseling centers. The methodological quality of included studies was assessed using the Cochrane risk-of-bias tool (33, 34).

Data were extracted from each eligible paper against a comprehensive data abstraction template with reference to the full article text. For the first review question (1), data were extracted on the delivery context, participant characteristics, study design, intervention details, outcome measures, and outcomes. The systematic review on the effectiveness of ASBI was part of the European Project BISTAIRS and can be read in detail elsewhere^1^ (35).

For the second and third review questions (2 and 3), data were extracted on any barriers to ASBI implementation identified in each effectiveness study. Further, in order to supplement the results for questions (2) and (3), additional guided searches were carried out focused around the additional questions of setting-specific implementation barriers and needs for further research. Compared to the report published in 2012, the present study (a) updated the search strategy; (b) used the Cochrane risk-of-bias tool for quality assessment; (c) expanded the research question by the analysis of barriers/facilitators; and (d) reviewed the need for future research in these settings. No statistical analyses or meta-analyses were conducted. Instead, the existing analyses reported in the articles reviewed were extracted systematically, with the findings reported in a structured narrative synthesis in response to the three overarching review questions.

## Results

### Evidence of effectiveness of ASBI in workplace settings

In this section, we provide an update of the results of our systematic review conducted in the framework for the EU project BISTAIRS^2^. Compared to the report published in 2012, the present study retrieved one additional article (36) in the workplace setting, resulting in 9 out of 3037 studies meeting our inclusion criteria (see Table 1). Key reasons for exclusion concerned, e.g., intervention characteristics (too long duration or general prevention), lack of effectiveness analyses, or inappropriate setting. The methodological quality varied due to study design, measurements, inclusion criteria, and analysis. Quality appraisal based on the Cochrane collaboration’s risk-of-bias tool revealed that most studies failed to describe in detail the approach to selection [random sequence generation (24, 36–40); allocation concealment (24, 37–42)] and performance biases [blinding of participants and personnel (24, 36–39, 41, 42)], resulting in the assessment of “unclear” in those areas. The random sequence generation of the study of Osilla et al. (41) was rated to have a “high risk-of-bias,” the reporting of Michaud et al. (36) was regarded as incomplete.

**Table 1:** **Evidence of effectiveness of ASBI, implementation barriers for ASBI, and future research needs for ASBI in workplace settings**.

Reference	Evidence of effectiveness of ASBI	Implementation barriers for ASBI	Future research needs for ASBI
Hermansson et al. (42)	Comparable reductions in all groups over time		Long-term effectiveness of alcohol interventions
Araki et al. (39)	Reductions in alcohol intake (g/day) for face-to face intervention	Low participation rates and group imbalances between control- and test group	
Anderson et al. (37)	Effect for number of drinking days, not for (peak) BAC	Low participation rates of hazardous and harmful drinkers	Filling the knowledge gap in relation to the cost-related outcomes of workplace ASBI
Osilla et al. (41)	Improvements for peak drinks/day and peak BAC; work performance improved in both groups	Lack of therapeutic work	Understand gender differences for implementing ASBIs in EAPs
Michaud et al. (36)	ASBI superiority for alcohol intake (g/week) and AUDIT mean score; reduction in AUDIT category in both groups	High rates of “lost” patients in follow-up	Evaluate important worksite cost-related outcomes, such as health care utilization, absenteeism rates, job performance ratings, turnover, and reported accidents
Doumas and Hannah (24)	Web-based and face-to face interventions both reduced peak consumption and weekend drinking		Tailoring an established model to young adults in the workplace
Walters and Woodall (38)	(Partly) significant reductions in drinking levels	Low participation rates of hazardous and harmful drinkers	
Matano et al. (40)	ASBI superiority in binge-drinking only for moderate drinkers	Potentially negative consequences of self-disclosure	
Hagger et al. (27)	ASBI superiority in units per week, both groups reduced binge drinking	Low participation rates of hazardous and harmful drinkers	Does present mental simulation intervention would have greater efficacy in a sample with hazardous levels of alcohol consumption and higher rates of binge-drinking occasions?

*AUDIT, alcohol use disorder identification test; BAC, blood alcohol concentration; EAP, employee assistance program*.

The majority of included studies were conducted in the USA (24, 37, 38, 40, 41), with a further three in Europe (27, 36, 42), with one in Japan (39). The company employment sector varied significantly, including organizations based in the transportation, food, and retail or manufacturing sectors. Some authors did not reveal specific company information due to privacy agreements with the companies. All companies were either large employers (about 1000 employees or more) or the participants were draw from several companies. The companies’ fields of activity and general description of the participants’ work (blue collar or white collar) varied between studies. Araki et al. (39) surveyed factory workers and some of the remaining studies were conducted in the service sector (24, 37, 42). However, the rest of the studies did not describe the workplace characteristics of their participants.

Recruitment of participants was either via management referral or company occupational health services. Methods for the identification of potentially harmful drinkers included adapted screening tools (e.g., AUDIT-C) or blood tests with unspecific or specific markers like carbohydrate-deficient-transferrin (CDT). All studies excluded participants with more intensive treatment needs due potential alcohol dependence (e.g., AUDIT score >19) or with severe health problems. The included studies tested face-to-face ASBI delivered by a trained counselor (27, 37, 41, 42), or web-based interventions, either alone (38, 40) or combined with a face-to-face approach (24, 39).

All except one study (42) showed significant reductions on alcohol consumption for brief interventions at least in some of their primary outcomes such as alcohol intake or numbers of drinking days. Araki et al. (39) observed a reduction of alcohol intake from 24.8 to 12.1 g ethanol/day. Anderson et al. (37) found a reduction of drinking days per week (from 2.39 to 1.95), and Osilla et al. (41) reported a significant reduction of peak drinks per occasion from 7.56 to 4.78 in the intervention group that received ASBI within an employee assistance program (EAP). Significant reduction in the AUDIT score after 12 months (6.59 vs. 7.55; *p* = 0.01) were found by Michaud et al. (36), but without showing significant effects in reducing hazardous drinking. The face-to-face plus website intervention of Doumas and Hannah (24) reduced the number of drinks per weekend from 2.42 to 1.87. Face-to-face ASBI was as effective as the stand-alone web-based intervention.

Three out of four studies, which used web-based interventions reported some positive effects (24, 38, 40). The participants in the intervention group of Walters and Woodall (38) decreased their alcohol consumption by 0.87 drinks per week (DPW), whereas those in the control group increased their consumption by 1.75 DPW. The website intervention scrutinized by Matano et al. (40) reduced binge drinking in participants with a moderate risk for alcohol problems by 48%, but due the inadequate sample size a further evaluation of treatment effects is not possible. The web-based interventions (web-based feedback and web-based feedback plus 15 min motivational interviewing) by Doumas and Hannah (24) show significant reductions of alcohol drinking within 30 days in young “high-risk” binge drinkers (defined by binge drinking at least once in the past 2 weeks). In contrast to these studies, Araki et al. (39) indicated that face-to-face educational interventions are more effective to increase the knowledge about and attitude toward drinking than a comparable email intervention. Noteworthy are the small response rates to web-based services, for instance, the website of Matano et al. (43) was visited by only 2.7% of all employees.

Finally, only the study by Hermansson et al. (42) found no superiority effects of ASBI compared to controls, but showed significant reductions in both groups. Most studies used short durations for follow-up of up to 6 months, only two choose follow-up assessment after 12 month (36, 42).

### Implementation barriers for ASBI in workplace settings

Effectiveness studies of ASBI in the workplace have mostly focused on the individual level obstacles experienced by both employers seeking to deliver alcohol prevention activities, and those employees who might benefit from such interventions. In contrast, there was no identified data illustrating organizational obstacles to routine ASBI delivery. In particular, as in other delivery settings, including PHC (44–46), the stigma associated with receiving an alcohol-related intervention impacts significantly on the implementation of ASBI in the workplace. Indeed, the reviewed studies suggest that this may be a reason for the low-participation rates of hazardous and harmful drinkers in this particular setting (37, 38). Employees may be anxious about participating in ASBI delivered at their workplace because of the potentially negative consequences of self-disclosure (43). Further, hazardous drinking is more prevalent in males, who are generally more inclined to reject therapeutic interventions for mental health conditions (47). In contrast to this, persons with a need of mental health service might more readily accept ASBI than those without (48, 49), which again might affect ASBI completion rates and outcome measures, and limit the generalizability of the results. Finally, the evidence also suggested that a lack of therapeutic work might be another reason for higher drop-out rates in ASBI groups (41).

### Future research needs for ASBI in workplace settings

The low participation and high-drop-out rates suggest that a clear need for further research both to explore the *acceptability* and *feasibility* of ASBI in workplace settings; and to address questions around the effective implementation of alcohol prevention strategies in different working environments. Further, there is an identified knowledge gap in relation to the cost-related outcomes of workplace ASBI [such as health care utilization, absenteeism rates, job performance ratings, turnover rates, and rate of work-related accidents (37)]; alongside the long-term effectiveness of alcohol interventions delivered in this setting (42).

In terms of the actual *effectiveness* of ASBI in the workplace, due to the limited number of RCTs in this field, it is not possible to identify under which circumstances ASBI is likely to effective, and or whether employees who work in a certain field would be more likely to benefit from specific ASBIs. We found no studies with workers from smaller companies and respective ASBI approaches for those employees are missing. In addition, most of our reviewed studies in workplace settings (five out of nine) were carried out in the United States, and thus, their outcomes cannot easily be transferred to the different and highly variable European health care and occupational health systems. There was also an absence of studies of workplace ASBI conducted in countries with a lower economic status.

### Evidence of effectiveness of ASBI in social services

In this section, we refer to the results of our systematic review conducted in the framework for the EU project BISTAIRS^2^, which can also be read in a critical commentary published in the BJSW (35). Six out of 1856 studies (seven publications) met our inclusion criteria (see Table 2). Reasons for exclusion included too long duration of intervention, lack of effectiveness analyses, or inappropriate setting. Two studies examine ASBI within homeless populations; two of which include homeless adolescents (50) and one study with homeless veterans (51). Another study has been conducted in a community-based drug and alcohol counseling center (52). In the criminal justice setting, we found three studies for inclusion, two of them conducted with participants arrested for driving while intoxicated (DWI) offenses (53, 54), and another among violent, alcohol-intoxicated offenders (55). These six studies show mixed results for the effectiveness of ASBI, and the heterogeneity of settings make it challenging to compare results. Compared to the report published in 2012, the updated search retrieved no additional studies to be included.

**Table 2:** **Evidence of effectiveness of ASBI, implementation barriers for ASBI, and future research needs for ASBI in social services**.

Reference	Evidence of effectiveness of ASBI	Implementation barriers for ASBI	Future research needs for ASBI
Peterson et al. (50)	No intervention effect on alcohol measures, but small effect on drug use	Low participation rates	To link ASBIs to others homeless services
Wain et al. (51)	Higher rates of treatment entry and completion		
Shakeshaft et al. (52)	Non-inferiority in drinking outcomes compared to CBT, better cost-effectiveness	Recruitment problems, as the majority did not know how to use a computer	Assessments of treatment outcome should measure actual behavior change, rather than perceptions of counseling alone
Wells-Parker and Williams (54)	Effect on DUI recidivism (60 months) for depressed subgroup	Social service providers might not feel responsible for alcohol-related interventions	
Brown et al. (53)	Reduction of risky drinking days in both groups	Low female participation rates	
Watt et al. (55)	Both groups improved in weekly units, no. of drinking days, and AUDIT score		Rather specialist referral, diagnostic assessments, and treatment than ASBI for high-bonded groups

*AUDIT, alcohol use disorder identification test; CBT, cognitive behavioral therapy; DUI, driving under the influence of alcohol*.

Peterson et al. (50) worked with homeless substance-using adolescents aged 14–19 years. Comparing brief motivational enhancement to one of two control groups (assessment only or assessment at follow-up), this study did not find any changes in alcohol measures (days of alcohol use, standard drink units, binge drinking), but demonstrated reductions in drug use (other than marijuana) at 1 month follow-up. In comparison, a study by Wain et al. (51) with alcohol-dependent homeless veterans measured the effectiveness of a single session of brief motivational interviewing upon treatment entry and completion. Treatment entry was significantly higher in the brief intervention group (95 vs. 71%; *p* = 0.017); and also length of stay, treatment completion, and graduation was higher, although these findings failed to reach significance (51). The study in a community-based drug and alcohol counseling center compared BI with the more intensive CBT. Here, the equal improvement of both BI and CBT participants in all drinking outcomes (weekly units, heavy drinking days, AUDIT scores) demonstrates a non-inferiority of ASBI, and the cost-effectiveness score was significantly better in the ASBI condition (52).

Among studies conducted in criminal justice settings, Watt et al. (51) conducted a study examining intervention with violent offenders comparing brief intervention against assessment only and found comparable reductions in both conditions for weekly units, number of drinking days, AUDIT scores, and heavy episodic drinking. Furthermore, no difference in recidivism rates could be determined during the 12-month follow-up period. However, significantly lower rates of injury (unintentional and self-harm) were reported in the brief intervention group (27.4 vs. 39.6%) (55). The two studies among DWI recidivists showed positive between-group findings on drinking levels favoring brief interventions, which approached significance (53, 54). Further, Wells-Parker and Williams (54) investigated differential effects on individuals with high- vs. low-depression scores (as measured by the sadness/depression subscale of the Mortimer-Filkins questionnaire). Although they failed to determine an overall superiority of adding two brief intervention sessions and a follow-up to standard treatment, rates of DWI recidivism were significantly lower among highly depressed participants receiving the extended brief intervention (16.7% extended brief intervention vs. 25.6% standard treatment) (54).

### Implementation barriers for ASBI in social services

As with alcohol prevention work delivered in workplace settings, research confirms that the participation rate in ASBI in social service and criminal justice settings is low, and the drop-out rate for follow-up is high. Further, compared to medical settings, which focus specifically on alcohol-related problems, the implementation of ASBI in these settings might result in additional personal challenges for social service providers, as they might not feel responsible for alcohol-related interventions (54). However, the lack of available evidence of ASBI in social services makes it challenging to draw firm conclusions in relation to the specific barriers and facilitators to their successful implementation in such settings. Moreover, the already identified heterogeneous nature of this setting, potentially suggests that approaches will need to be carefully tailored to the specific needs of different delivery contexts.

For example, looking at Peterson’s study with homeless adolescents (42), given the multiple social, psychological, and health problems often experienced by homeless adolescents, one may conclude that a brief intervention of around 30 min is simply not sufficient to intervene with such needs. Moreover, instability and transience characterize the lives of homeless youth, resulting in intensive and sustained intervention being hard to achieve. In addition, the study by Watt et al. showed that alcohol-dependent clients are highly prevalent in services of criminal justice systems (55). More than one-third of the sample scored >20 in AUDIT, thus, exceeding the indicative cut-off points for alcohol dependence. As such, those clients need specialist referral, diagnostic assessments, and treatment, rather than ASBI. Both these examples, suggest that a stepped-care approach of the type discussed in relation to the Wells-Parker and Williams (54) study above, is likely to be an important consideration in designing ASBI implementation strategies within social service settings.

### Future research needs for ASBI in social services

Of all the potential delivery contexts for ASBI, the evidence base around social service settings remains arguably in its infancy. While it may well be possible to capitalize on the substantial progress made in this research field in other settings (and in particular in ASBI), the low-participation/high-drop-out rates and complex client needs suggest that a strong need for further work to explore the feasibility and acceptability of ASBI work in this varied and challenging delivery context.

## Discussion

In stark contrast to the robust and comprehensive literature supporting their effectiveness in PHC, the ASBI research field in non-medical settings paints a far more complex, patchy, and varied picture of what works best, in which contexts, and with whom. Since our previously published BISTAIRS project report (REF), this picture has changed little, with only one additional study retrieved in the search update. As such, the evidence base for ASBI in non-medical settings remains sparse.

While the results of this review provide some encouraging support for ASBI delivery in workplace settings, it also highlights the fact that there has been little attention paid to research based in this context to date, despite this being where millions of working-age adults spend most of their day. Currently, the development of ASBI workplace approaches has been restricted to occupational health services in large factories, and therefore, little is known about whether such strategies would be transferable to smaller organizations, or to businesses outside the manufacturing or construction sectors. Nevertheless, although the evidence does not yet suggest any clear recommendation for a widespread implementation of workplace ASBI, occupational health services could consider offering brief advice to employees who are considered as drinking in a risky or potentially harmful way. A useful toolkit and manual has been issued by the European workplace and alcohol (EWA) project (56). Further, the evidence does emphasize the importance of the existence of comprehensive alcohol at work policy, embedded within overall healthy living policies and actions at the workplace, that take into account the structural and working environments that increase risky drinking in the first place (57). Results of the Swedish Risk Drinking Project, which implemented tailored training courses around ASBI in a large number of primary, maternal, and occupational health services, demonstrated improvements in knowledge, self-efficacy, and alcohol-preventive activity in occupational health services, especially in nurses, who were afforded a key role in the project (58).

The evidence base for ASBI in social services is essentially non-existent, and although some reviews (59) and some trials (60) have included social service settings, it is difficult to identify a clear positive impact of brief advice programs. The UK criminal justice system – screening and intervention program for sensible drinking (SIPS) trial found evidence for an impact of receipt of a patient information leaflet, brief advice, and brief lifestyle counseling, with no differences between the three interventions (61). Thus, because of the paucity of evidence, rather than suggesting comprehensive delivery of roll-out of brief advice programs in social service settings, it might be more beneficial at this stage to gather further evidence as to the acceptability and feasibility of ASBI in social service settings, generating useful system readiness data, until more evidence for effectiveness is gathered.

In particular, for example, future studies need to consider what setting-specific differences exist (in terms of client–patient target groups, institutional characteristics, or acceptance among professionals), and to assess how these differences might influence the deliverability, acceptance, and potential effectiveness of ASBI. Receiving and delivering alcohol interventions in the types of non-medical settings described in this paper entails a range of client–provider relationships and expectations that are arguably not easily comparable to those evident in generalist medical settings. In PHC, for example, individual patients often build up long-term, positive relationships with their GP and practice nurse (62), and (crucially) are generally motivated to enter into such relationships for primarily health-related reasons. ASBI strategies that prove successful in PHC, therefore, may not be appropriate for implementation in the workplace, where employer and employee are necessarily financially committed to each other. However, in the framework of occupational health services, a setting, which is more comparable to PHC, this barrier might be reduced, as occupational health staff is supposed to keep confidentiality. Another difficulty to the acceptance of ASBI may arise in criminal justice systems, where offenders are engaged in an involuntary, legally binding relationship with their probation workers as a result of “deviant” behavior.

Further, and in particular, in respect of ASBI in social service settings, one might also question whether a focus on drinking reductions is a realistic and achievable first-line goal for all target groups that social service professionals might come into contact with. The studies with homeless people (who generally have more needs and numerous impairments other than alcohol abuse), suggest that brief approaches may be unlikely to reduce drinking levels in certain patient populations (50, 51, 63), but that other factors might be successfully addressed, such as rates of entry in addiction treatment (51) and service utilization (63). Further, the results of Wells-Parker and Williams (54) in DWI offenders with high rates of depression and low self-efficacy, but high willingness to reduce consumption suggest that additional motivational components might not be necessary for all risky drinkers to achieve drinking reductions, but they may be of relevance for particular subgroups. Providing extended interventions only to those in need, is in line with stepped-care approaches (64). For certain client groups, BI approaches might thus more serve as a “door-opener,” in the sense of enabling referral to other services, and should not be seen as a tool, which directly influences the amount of drinking.

At the same time, and while recognizing the heterogeneous nature of the social services evidence base, it was notable that in all except one study in these settings (homeless youth), control groups achieved comparable reductions in their drinking levels over time. This is in line with previous findings from ASBI studies in the medical field. For example, drinking reductions ranging between 10 and 40% among participants in control groups were shown in reviews by Jenkins et al. (65) and Bernstein et al. (66). A further review by McCambridge and Kypri (67) comparing longer vs. shorter (or no) assessment found reductions in weekly consumption levels attributable to interview procedures. In addition, the recent SIPS trials, conducted in primary care practices, could not determine a significant additional benefit of brief advice or lifestyle counseling over and above the provision of short personalized feedback and provision of a leaflet (68). This non-inferiority of “control” conditions might suggest that the implementation of any kind of very brief alcohol interventions may be of value, even in these challenging settings.

In conclusion, therefore, the overriding message is that “more research is needed,” and in particular, that there is a strong need for more robust ASBI trials in non-medical settings in order to address the identified knowledge gaps on obstacles and difficulties in ASBI implementation in these settings. In tandem with outcome assessments, information on the acceptability and feasibility of ASBI in their various forms are needed to provide data on the system readiness for workplace and social care settings, rather than focusing solely on demonstrating ASBI effectiveness. However, given the large existing evidence base for ASBI in PHC and other health settings, which has taken decades to accrue, it is nevertheless to be hoped that alcohol prevention work in occupational and social service settings might gain from this substantial body of knowledge in order accelerate the evaluative process and achieve the potential benefits for clients and employees in a far shorter time-frame.

## Conflict of Interest Statement

The authors declare that the research was conducted in the absence of any commercial or financial relationships that could be construed as a potential conflict of interest.

## Footnotes

^1^http://bistairs.eu/material/BISTAIRS_WP4_evidence_report.pdf

^2^www.bistairs.eu

## References

[1] Rehm J Room R Monteiro M Gmel G Graham K Rehn N Alcohol as a risk factor for global burden of disease. Eur Addict Res (2003) 9:157–64 10.1159/000072222 12970584

[2] Lim SS Vos T Flaxman AD Danaei G Shibuya K Ezzati M A comparative risk assessment of burden of disease and injury attributable to 67 risk factors and risk factor clusters in 21 regions, 1990-2010: a systematic analysis for the global burden of disease study 2010. Lancet (2012) 380(9859):2224–60 10.1016/S0140-6736(12)61766-8 23245609 PMC4156511

[3] NAO. Reducing Alcohol Harm: Health Services in England for Alcohol Misuse. London: National Audit Office (2008).

[4] O’Donnell A Anderson P Newbury-Birch D Schulte B Schmidt C Reimer J The impact of brief alcohol interventions in primary healthcare: a systematic review of reviews. Alcohol Alcohol (2014) 49(1):66–78 10.1093/alcalc/agt170 24232177 PMC3865817

[5] NICE. Alcohol-Use Disorders: Preventing Harmful Drinking. NICE Public Health Guidance 24. Manchester: National Institute for Health and Clinical Excellence (2010).

[6] Nilsen P Baird J Mello M Nirenberg T Woolard R Bendtsen P A systematic review of emergency care brief alcohol interventions for injury patients. J Subst Abuse Treat (2008) 35:184–201 10.1016/j.jsat.2007.09.008 18083321

[7] McQueen J Howe T Allan L Mains D Hardy V Brief interventions for heavy alcohol users admitted to general hospital wards. Cochrane Database Syst Rev (2011) (8):CD005191 10.1002/14651858.CD005191.pub3 21833953 PMC10600352

[8] Forrester D Harwin J Parental substance misuse and child care social work: findings from the first stage of a study of 100 families. Child Fam Soc Work (2006) 11:325–35 10.1111/j.1365-2206.2006.00415.x

[9] Hughes K Anderson Z Morleo M Bellis MA Alcohol, nightlife and violence: the relative contributions of drinking before and during nights out to negative health and criminal outcomes. Addiction (2008) 103(1):60–5 10.1111/j.1360-0443.2007.02030.x 17996008

[10] Anderson P Chisholm D Fuhr D Effectiveness and cost-effectiveness of policies and programmes to reduce the harm caused by alcohol. Lancet (2009) 373:2234–46 10.1016/S0140-6736(09)60744-3 19560605

[11] Rehm J The risks associated with alcohol use and alcoholism. Alcohol Res Health (2011) 34(2):135–43 22330211 PMC3307043

[12] Bliss DL Pecukonis E Screening and brief intervention practice model for social workers in non-substance-abuse practice settings. J Soc Work Pract Addict (2009) 9:21–40 10.1080/15332560802646604

[13] Karoll BR Applying social work approaches, harm reduction, and practice wisdom to better serve those with alcohol and drug use disorders. J Soc Work (2010) 10(3):263–81 10.1177/1468017310363635

[14] Smith MJW Whitaker T Weismiller T Social workers in the substance abuse treatment field: a snapshot of service activities. Health Soc Work (2006) 31(2):109–15 10.1093/hsw/31.2.109 16776028

[15] Weaver T Madden P Charles V Stimson G Renton A Tyrer P Comorbidity of substance misuse and mental illness in community mental health and substance misuse services. Br J Psychiatry (2003) 183:304–13 10.1192/bjp.183.4.304 14519608

[16] Dar K Alcohol use disorders in elderly people: fact or fiction? Adv Psychiatr Treat (2006) 12(3):173–81 10.1192/apt.12.3.173

[17] Schilling R Dornig K Lungren L Treatment of heroin dependence: effectiveness, costs, and benefits of methadone maintenance. Res Soc Work Pract (2006) 16(1):48–56 10.1177/1049731505277059

[18] Babor TF Ritson EB Hodgson T Alcohol-related problems in the primary health care setting: a review of early intervention strategies. Br J Addict (1986) 81:23–46 10.1111/j.1360-0443.1986.tb00291.x 3457598

[19] Cook RF Back AS Trudeau J Preventing alcohol use problems among blue-collar workers: a field test of the working people program. Subst Use Misuse (1996) 31(3):255–75 10.3109/10826089609045812 8834262

[20] Mangione TW Howland J Amick B Cote J Lee M Bell N Employee drinking practices and work performance. J Stud Alcohol (1999) 60(2):261–70 10091965 10.15288/jsa.1999.60.261

[21] Hermansson U Helander A Brandt L Huss A Ronnberg S The alcohol use disorders identification test and carbohydrate-deficient transferrin in alcohol-related sickness absence. Alcohol Clin Exp Res (2002) 26(1):28–35 10.1111/j.1530-0277.2002.tb02428.x 11821651

[22] Bacharach SB Bamberger P Biron M Alcohol consumption and workplace absenteeism: the moderating effect of social support. J Appl Psychol (2010) 95(2):334–48 10.1037/a0018018 20230073 PMC2903009

[23] Aichberger MC Yesil R Rapp MA Schlattmann P Temur-Erman S Bromand Z Surveying migrant populations – methodological considerations: an example from Germany. Int J Cult Ment Health (2012) 6:1–15 10.1080/17542863.2011.642981

[24] Doumas DM Hannah E Preventing high-risk drinking in youth in the workplace: a web-based non-native feedback program. J Subst Abuse Treat (2008) 34(3):263–71 10.1016/j.jsat.2007.04.006 17600650

[25] Batts KR Grabill TC Galvin DM Schlenger WE Contextual and Other Factors Related to Workplace-Based Substance Abuse Prevention and Early Intervention for Adolescents and Young Adults. Washington, DC: US Department of Health and Human Services (2005).

[26] Dickman F Emener WG Employee assistance programs: basic concepts, attributes and an evaluation. Pers Adm (1982) 27(8):55–62 10258031

[27] Hagger MS Lonsdale A Chatzisarantis NLD Effectiveness of a brief intervention using mental simulations in reducing alcohol consumption in corporate employees. Psychol Health Med (2011) 16(4):375–92 10.1080/13548506.2011.554568 21749236

[28] Roman PM Blum TC Alcohol: a review of the impact of worksite interventions on health and behavioral outcomes. Am J Health Promot (1996) 11(2):136–49 10.4278/0890-1171-11.2.136 10163599

[29] Fouriaud C Jacquinet-Salord M Mahé IR MJ TL Occupational medicine and general prevention: results of an epidemiological survey among 8203 workers. Archives des maladies professionnelles de médecine du travail et sécurité sociale (1991) 52(5):333–7

[30] Rehm J Roerecke M Reduction of drinking in problem drinkers and all-cause mortality. Alcohol Alcohol (2013) 48(4):509–13 10.1093/alcalc/agt021 23531718

[31] Edwards G Arif A Hadgson R Nomenclature and classification of drug- and alcohol-related problems: a WHO memorandum. Bull World Health Organ (1981) 59(2):225–42 6972816 PMC2396054

[32] World Health Organization. International Classification of Diseases. Geneva: American Psychiatric Publishing (1992).

[33] Higgins J Green S, editors. . Cochrane Handbook for Systematic Reviews of Interventions Version 5.1.0 [Updated March 2011]. The Cochrane Collaboration (2011). Available from: http://www.cochrane-handbook.org

[34] Higgins JP Altman DG Gotzsche PC Juni P Moher D Oxman AD The Cochrane collaboration’s tool for assessing risk of bias in randomised trials. BMJ (2011) 343:d5928 10.1136/bmj.d5928 22008217 PMC3196245

[35] Schmidt CS McGovern R Schulte B O’Donnell A Lehmann K Kuhn S Brief alcohol interventions in social service and criminal justice settings – a critical commentary. Br J Soc Work (2014) (in press).

[36] Michaud P Kunz V Demortiere G Lancrenon S Carre A Menard C Efficiency of brief interventions on alcohol-related risks in occupational medicine. Glob Health Promot (2013) 20(2 Suppl):99–105 10.1177/1757975913483339 23678504

[37] Anderson BK Larimer ME Problem drinking and the workplace: an individualized approach to prevention. Psychol Addict Behav (2002) 16(3):243–51 10.1037/0893-164X.16.3.243 12236459

[38] Walters ST Woodall W Mailed feedback reduces consumption among moderate drinkers who are employed. Prev Sci (2003) 4:287–94 10.1023/A:1026024400450 14599000

[39] Araki I Hashimoto H Kono K Matsuki H Yano E Controlled trial of worksite health education through face-to-face counseling vs. e-mail on drinking behavior modification. J Occup Health (2006) 48(4):239–45 10.1539/joh.48.239 16902267

[40] Matano RA Koopman C Wanat SF Winzelberg AJ Whitsell SD Westrup D A pilot study of an interactive web site in the workplace for reducing alcohol consumption. J Subst Abuse Treat (2007) 32(1):71–80 10.1016/j.jsat.2006.05.020 17175400

[41] Osilla KC Zellmer SP Larimer ME Neighbors C Marlatt GA A brief intervention for at-risk drinking in an employee assistance program. J Stud Alcohol Drugs (2008) 69(1):14–20 18080060 10.15288/jsad.2008.69.14

[42] Hermansson U Helander A Brandt L Huss A Ronnberg S Screening and brief intervention for risky alcohol consumption in the workplace: results of a 1-year randomized controlled study. Alcohol Alcohol (2010) 45(3):252–7 10.1093/alcalc/agq021 20406791

[43] Matano RA Koopman C Wanat SF Whitsell SD Borggrefe A Westrup D Assessment of binge drinking of alcohol in highly educated employees. Addict Behav (2003) 28(7):1299–310 10.1016/S0306-4603(02)00248-4 12915170

[44] Aira M Kauhanen J Larivaara P Rautio P Factors influencing inquiry about patients’ alcohol consumption by primary health care physicians: qualitative semi-structured interview study. Fam Pract (2003) 20(3):270–5 10.1093/fampra/cmg307 12738695

[45] Moriarty HJ Stubbe MH Chen L Tester RM Macdonald LM Dowell AC Challenges to alcohol and other drug discussions in the general practice consultation. Fam Pract (2012) 29(2):213–22 10.1093/fampra/cmr082 21987374 PMC3312113

[46] Shaw S Cartwright A Spratley T Harwin J Responding to Drinking Problems. Baltimore: University Park Press (1978).

[47] Chan KK Neighbors C Marlatt GA Treating addictive behaviors in the employee assistance program: implications for brief interventions. Addict Behav (2004) 29(9):1883–7 10.1016/j.addbeh.2004.05.004 15530733

[48] Neighbors C Palmer RS Larimer ME Interest and participation in a college student alcohol intervention study as a function of typical drinking. J Stud Alcohol (2004) 65(6):736–40 15700511 10.15288/jsa.2004.65.736

[49] Blum TC Roman PM A description of clients using employee assistance programs. Alcohol Health Res World (1992) 16:120–8

[50] Peterson PL Baer JS Wells EA Ginzler JA Garrett SB Short-term effects of a brief motivational intervention to reduce alcohol and drug risk among homeless adolescents. Psychol Addict Behav (2006) 20(3):254–64 10.1037/0893-164X.20.3.254 16938063

[51] Wain RM Wilbourne PL Harris KW Pierson H Teleki J Burling TA Motivational interviewing improves treatment entry in homeless veterans. Drug Alcohol Depend (2011) 115(1–2):113–9 10.1016/j.drugalcdep.2010.11.006 21145181

[52] Shakeshaft AP Bowman JA Burrows S Doran CM Sanson-Fisher RW Community-based alcohol counselling: a randomized clinical trial. Addiction (2002) 97(11):1449–63 10.1046/j.1360-0443.2002.00199.x 12410785

[53] Brown TG Dongier M Ouimet MC Tremblay J Chanut TF Legault L Brief motivational interviewing for DWI recidivists who abuse alcohol and are not participating in DWI intervention: a randomized controlled trial. Alcohol Clin Exp Res (2010) 34(2):292–301 10.1111/j.1530-0277.2009.01092.x 19930236

[54] Wells-Parker E Williams M Enhancing the effectiveness of traditional interventions with drinking drivers by adding brief individual intervention components. J Stud Alcohol (2002) 63(6):655–64 12529065 10.15288/jsa.2002.63.655

[55] Watt K Shepherd J Newcombe R Drunk and dangerous: a randomised controlled trial of alcohol brief intervention for violent offenders. J Exp Criminol (2008) 4:1–19 10.1007/s11292-007-9048-7

[56] Dawson J Rodriguez-Jareño M Segura L Colom J European Workplace and Alcohol Toolkit for Alcohol-Related Interventions in Workplace Settings. Barcelona: Department of Health of the Government of Catalonia (2013).

[57] Anderson P Alcohol and the Workplace. Barcelona: World Health Organisation, Regional Office for Europe (2012). Available from: http://www.euro.who.int/__data/assets/pdf_file/0009/191367/8-Alcohol-and-the-workplace.pdf

[58] Nilsen P Wahlin S Heather N Implementing brief interventions in health care: lessons learned from the Swedish risk drinking project. Int J Environ Res Public Health (2011) 8(9):3609–27 10.3390/ijerph8093609 22016706 PMC3194107

[59] Moyer A Finney JW Swearingen CE Vergun P Brief interventions for alcohol problems: a meta-analytic review of controlled investigations in treatment-seeking and non-treatment-seeking populations. Addiction (2002) 97(3):279–92 10.1046/j.1360-0443.2002.00018.x 11964101

[60] Babor T Grant M Project on Identification and Management of Alcohol-Related Problems. Report on Phase II: A Randomized Clinical Trial of Brief Interventions in the Primary Health Care Setting. Geneva: World Health Organization (1992).

[61] Newbury-Birch D Kaner E Deluca P Coulton S A randomized controlled trial of different methods of alcohol screening and brief intervention in routine probation settings: 12-month outcomes. Addict Sci Clin Pract (2012) 7(Suppl 1):A82 10.1186/1471-2458-9-418 19922618 PMC2784463

[62] Lock C Screening and brief alcohol interventions: what, why, who, where and when? A review of the literature. J Subst Abuse (2004) 9(2):91–101 10.1080/14659890410001665096

[63] Baer JS Grarrett SB Beadnell B Wells EA Peterson PL Brief motivational interventions with homeless adolescents: evaluating effects on substance use and service utilization. Psychol Addict Behav (2007) 21(4):582–6 10.1037/0893-164X.21.4.582 18072842

[64] Sobell MB Sobell LC Stepped care as a heuristic approach to the treatment of alcohol problems. J Consult Clin Psychol (2000) 68(4):573–9 10.1037//0022-006x.68.4.573 10965632

[65] Jenkins RJ McAlaney J McCambridge J Change over time in alcohol consumption in control groups in brief intervention studies: systematic review and meta-regression study. Drug Alcohol Depend (2009) 100(1–2):107–14 10.1016/j.drugalcdep.2008.09.016 19041196

[66] Bernstein JA Bernstein E Heeren TC Mechanisms of change in control group drinking in clinical trials of brief alcohol intervention: implications for bias toward the null. Drug Alcohol Rev (2010) 29(5):498–507 10.1111/j.1465-3362.2010.00174.x 20887573

[67] McCambridge J Kypri K Can simply answering research questions change behaviour? Systematic review and meta analyses of brief alcohol intervention trials. PLoS One (2011) 6(10):e23748 10.1371/journal.pone.0023748 21998626 PMC3187747

[68] Kaner E Bland M Cassidy P Coulton S Dale V Deluca P Effectiveness of screening and brief alcohol intervention in primary care (SIPS trial): pragmatic cluster randomised controlled trial. BMJ (2013) 346:e8501 10.1136/bmj.e8501 23303891 PMC3541471

